# Comparative transcriptomic and proteomic analyses provide insights into functional genes for hypoxic adaptation in embryos of Tibetan chickens

**DOI:** 10.1038/s41598-020-68178-w

**Published:** 2020-07-08

**Authors:** Ying Zhang, Xiaotong Zheng, Yawen Zhang, Hongliang Zhang, Xuyuan Zhang, Hao Zhang

**Affiliations:** National Engineering Laboratory for Animal Breeding, Beijing Key Laboratory for Animal Genetic Improvement, College of Animal Science and Technology, China Agricultural University, Beijing, China

**Keywords:** Animal breeding, Sequencing, Gene expression

## Abstract

The Tibetan chicken is a unique breed that has adapted to the high-altitude hypoxic conditions of the Tibetan plateau. A number of positively selected genes have been reported in these chickens; however, the mechanisms of gene expression for hypoxia adaptation are not fully understood. In the present study, eggs from Tibetan and Chahua chickens were incubated under hypoxic and normoxic conditions, and vascularization in the chorioallantoic membrane (CAM) of embryos was observed. We found that the vessel density index in the CAM of Tibetan chickens was lower than in Chahua chickens under hypoxia conditions. Transcriptomic and proteomic analyses of CAM tissues were performed in Tibetan and Chahua chicken embryos under hypoxic incubation using RNA-Seq and iTRAQ. We obtained 160 differentially expressed genes and 387 differentially expressed proteins that were mainly enriched in angiogenesis, vasculature development, blood vessel morphogenesis, blood circulation, renin-angiotensin system, and HIF-1 and VEGF signaling pathways. Twenty-six genes involved in angiogenesis and blood circulation, two genes involved in ion transport, and six genes that regulated energy metabolism were identified as candidate functional genes in regulating hypoxic adaptation of chicken embryos. This research provided insights into the molecular mechanism of hypoxia adaptation in Tibetan chickens.

## Introduction

Owing to the power of evolution and selection, humans as well as other animals native to high altitude areas cause inheritable changes in morphological structures, physiological and biochemical characteristics, and molecular regulatory pathways to survive in hypoxic environments. The Tibetan chicken is a highland native breed that inhabits at altitudes of 2,200–4,100 m within the Qinghai-Tibet Plateau and shows high adaptability in hypoxic environments^1^, and exhibiting good performances in embryonic survival and reproduction^2,3^. In fact, the embryo mortality rate of Tibetan chickens is markedly lower than that of lowland chickens under hypoxic incubation^4, 5^. Hence, Tibetan chicken embryos are useful models for investigating the genetic mechanisms of hypoxic adaptation. Alternatively, the Chahua chicken is domesticated from the red jungle fowl, which is a low-altitude typical local chicken breed in Yunnan Province^6^.

Several genomic diversity analyses have been conducted to determine the functional genes of adaptation to high-altitude in Tibetans^7,8^, Tibetan pigs^9,10^, Tibetan grey wolves^11^, ground tits^12^, Tibetan sheep^13^, yaks^14^, Tibetan antelopes^15^, Tibetan mastiffs^16^, and Tibetan cashmere goats^17^, as well as Tibetan chickens^18,19^. The genetic mechanisms of adaptation through a complex trait involving multi-gene expression are not fully understood. Developing transcriptomic and proteomic profiles allows for the identification of genes involved in the regulation of hypoxic adaptation at the mRNA and protein expression levels. RNA sequencing (RNA-Seq), as a transcript quantification technology, provides a more precise measurement of transcripts levels and isoforms compared to alternative approaches, and thus has been widely and successfully applied in various species^20,21^. Similarly, proteomics is considered to be a reliable and repeatable high-throughput method for understanding biological processes, and relative and absolute quantitative isobaric labeling (iTRAQ) as second generation gel-free proteomic analysis has been shown to more accurately quantify protein levels^22,23^. Integrating transcriptome and proteome analyses could provide insights into key functional genes and their regulatory mechanisms underlying hypoxic adaptation in Tibetan chicken embryos.

Chorioallantoic membrane (CAM), a respiratory and circulatory organ in chicken embryonic development, contains numerous blood vessels^24,25^. The CAM is a highly vascularized extraembryonic membrane that mediates gas (oxygen) exchange and nutrients during embryonic chick development^26^. Hypobaric hypoxia can induce vascular density and increases in the CAM to improve oxygen uptake and transport^27^. Under normoxia incubation, angiogenesis in chicken CAM gradually increases from day 4, and the vascular density reaches a plateau at day 14 of incubation^28^. Both mass and vascular network of CAM begin to rapidly grow at day 11 of incubation, which are key stages for CAM structure and function during chicken embryonic development. Therefore, genome-wide gene expressions in the CAM tissue at day 11 can be used to analyze functional genes that regulate CAM development and identify potential genes for hypoxic adaptation in the Tibetan chicken embryo.

In this study, we performed a comparative analysis of the transcriptomic and proteomic profiles of CAM tissues in Tibetan and Chahua chickens incubated under hypoxic environments using RNA-Seq and iTRAQ technologies. The object was to identify key functional genes and pathways involved in the hypoxic adaptation of the chicken embryo.

## Results

### Vascularization in CAM

**Figure 1:**
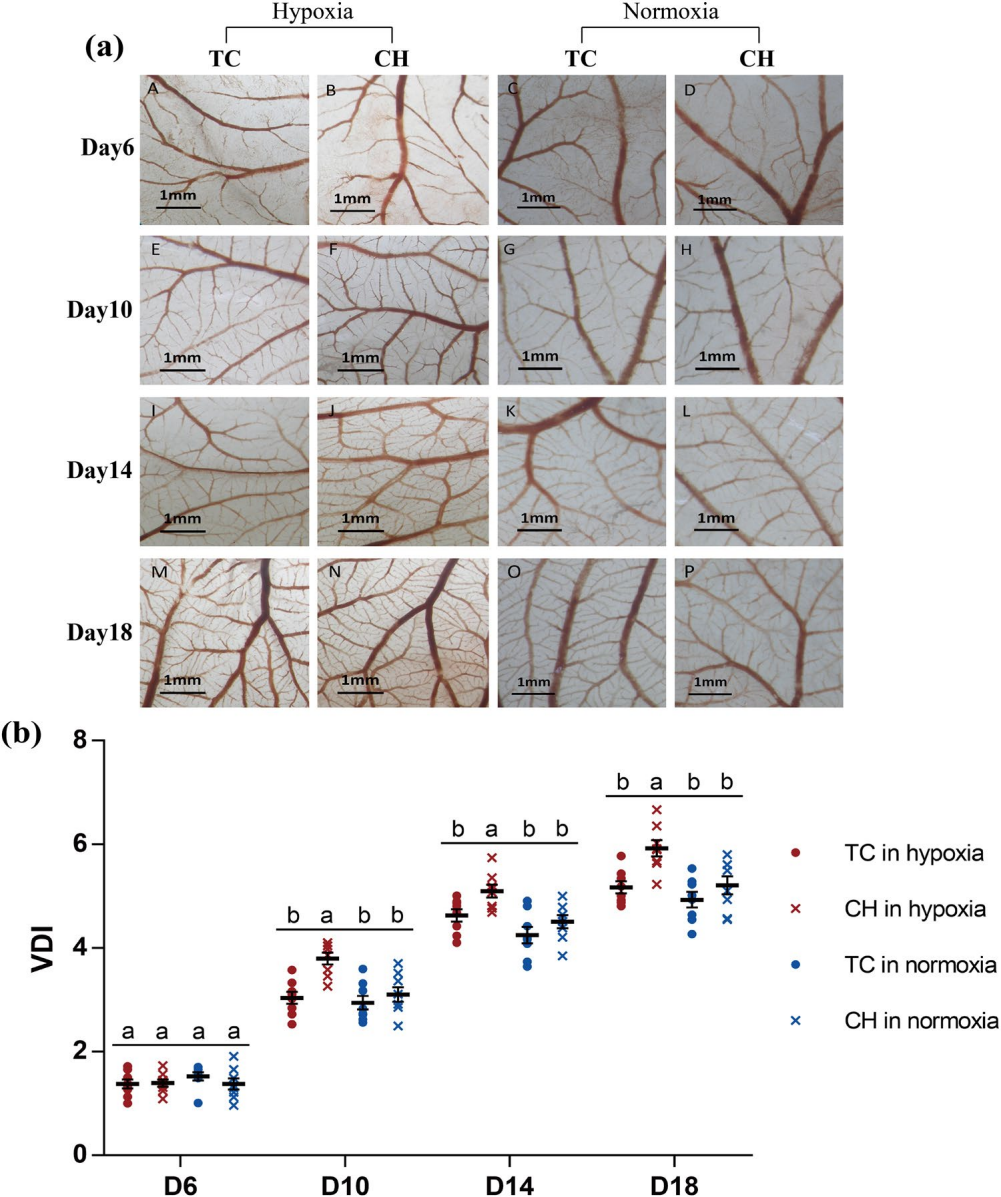
Vascularity of CAM during incubation. (**a**) Panels A to P are representative photomicrographs for days 6, 10, 14, and 18 of incubation, showing CAMs that had been exposed to hypoxic and normoxic conditions; (**b**) the quantitative comparisons of VDI under hypoxic and normoxic conditions, all values are represented as the mean ± standard error. Values labeled with the same letter (a, b) do not differ significantly; values labeled with different letters show significant differences at *P* < 0.05. Red color represents chickens incubated in hypoxic condition and blue color represent chickens incubated in normoxic condition. *CAM* chorioallantoic membrane, *VDI* vascular density index, *TC* Tibetan chicken, *CH* Chahua chicken.

The eggs of Tibetan chicken (TC) and Chahua chicken (CH) were incubated in the hypoxic incubator (13% ± 0.2% O_2_) and normoxic incubator (21% ± 0.2% O_2_). The vessel density index (VDI) value in CAM of embryos were performed on days 6, 10, 14, and 18 of incubation. We found that the VDI levels in CAM increased rapidly from days 6 to 18 during embryonic development (Fig. 1). On day 6 of incubation, the CAM covered a small area and the VDI was not significant different between the hypoxic and normoxic incubations in both the TC and CH, which might be due to the hypoxic effects on angiogenesis that were not enough to observe on VDI at the early embryonic developmental stage. After the 10th day, the VDI of CAM under hypoxic incubation was significantly higher than that of normoxia in Chahua chickens (*P* < 0.05). However, the difference in VDI between the hypoxic and normoxic incubation was not significant in TC (*P* > 0.05). Under hypoxic incubation, the VDI of the TC was significantly lower than the CH (*P* < 0.05) and the response of the hypoxia on angiogenesis was blunted, which might be an adaptation characteristic of CAM vessels in the Tibetan chickens (Fig. 1b, Table S1).

## Summary of transcriptomic profiles of chicken CAM tissues

For each RNA-Seq sample, we obtained 51.4–91.1 M clean reads, in which 74.30–78.90% was mapped to the chicken reference genome (Table S2). The majority of the mapped reads were distributed in annotated genic body regions, in which 66.85–69.02% were located in annotated exons and 9.72–12.03% in introns (Fig. S1).

**Figure 2:**
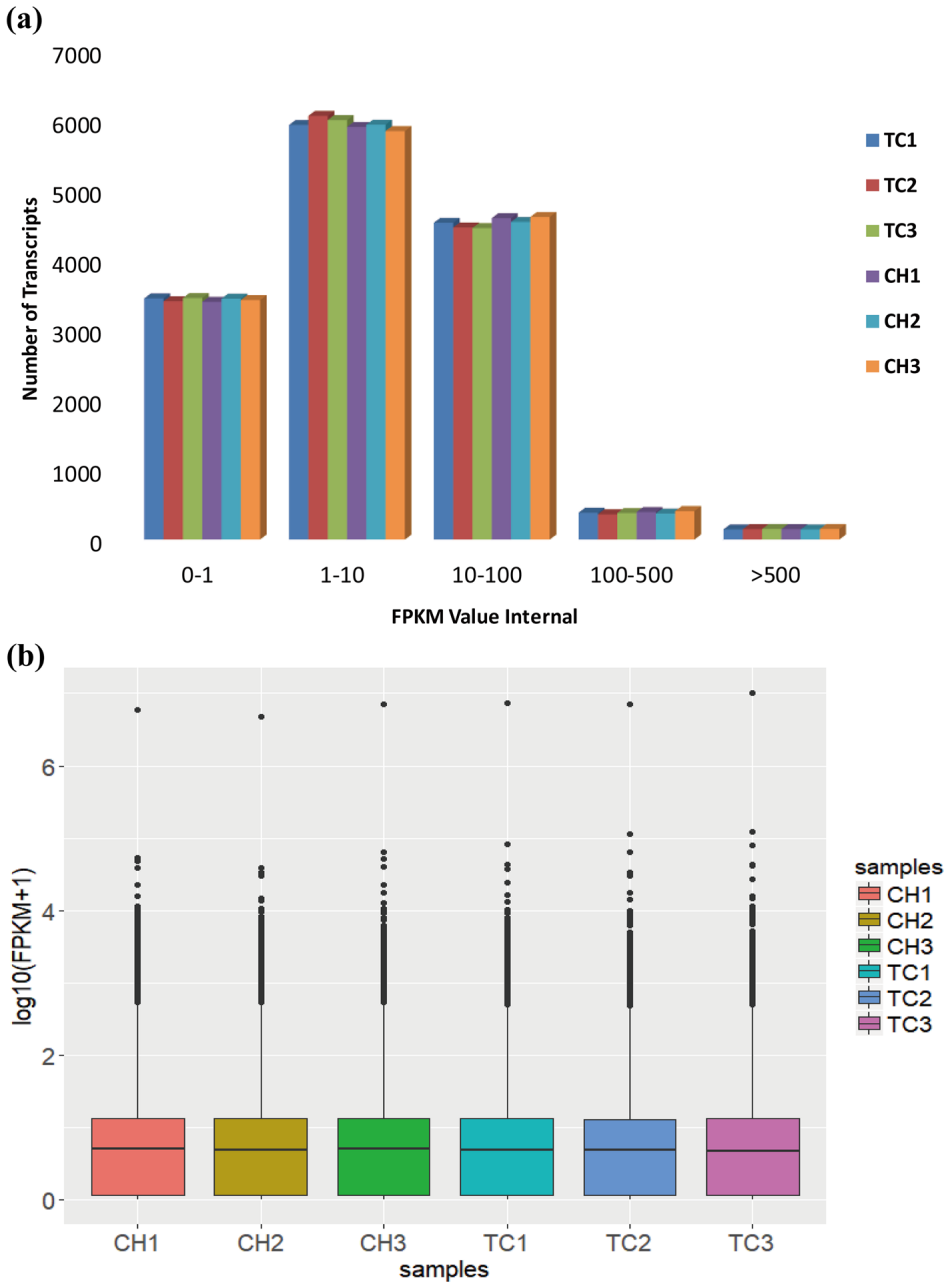
Distribution of positive expression genes in the chicken embryos. (**a**) The number of detected genes with different expression levels against the range of fragments per kilobase of exon length million mapped reads (FPKM) values. (**b**) Distributions of expression values of six samples. The box-and-whisker plots show log10 (FPKM + 1) of each gene from the six sets of RNA-Seq data. The black line in the box represents the median. *TC* Tibetan chicken, *CH* Chahua chicken.

In total, 14,869 expressed genes were detected in CAM tissues, in which 14,349 genes shared expression in TC and CH (Fig. S2). The fragments per kilobase of transcript per million (FPKM) values of expressed genes in each sample were largely between 1–10 and 10–100 (Fig. 2a), and the values of distribution were similar among the six samples (Fig. 2b). The clustered heat maps with FPKM values for all overlapping genes showed three replicate samples within groups clustered together (Fig. S3).

### Identification and functional analysis of differentially expressed genes (DEGs)

**Figure 3:**
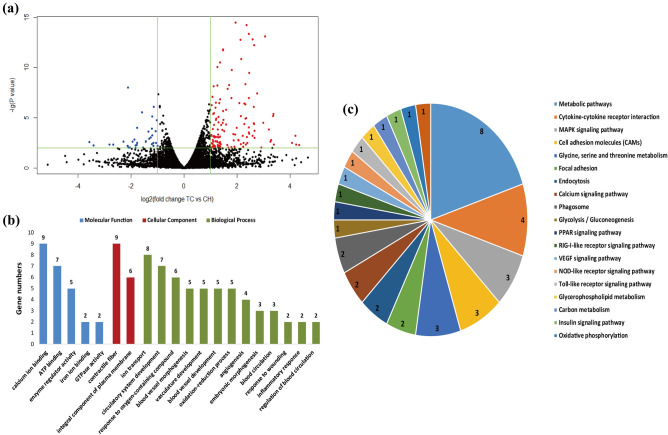
Identification and functional analysis of DEGs. (**a**) Volcano plot displaying DEGs between TC and CH. Up-regulated and down-regulated genes are shown in red and blue, respectively. Black dots represent genes with similar expression levels. (**b**) GO enrichment analysis of DEGs between TC and CH. The x-axis shows GO enrichment terms, and the y-axis represents gene numbers. (**c**) KEGG-enriched pie of DEGs between TC and CH^56^. The different colors represent pathway enrichment terms. Genes numbers enriched in the KEGG pathways are marked on the pie chart. *TC* Tibetan chicken, *CH* Chahua chicken.

A total of 160 DEGs (126 up-regulated and 34 down-regulated) were identified between TC and CH groups (Fig. 3a, Table S3). The 160 DEGs were classified into two categories for the six samples, and the heat tones of FPKM values showed the expressed quantity of the DEGs have good repeatability within groups (Fig. S4).

The DEGs were enriched in critical gene ontology (GO) terms that included blood vessel morphogenesis, vasculature development, angiogenesis, blood circulation, inflammatory response, ion transport, ATP binding, and other related biological processes (Fig. 3b, Table S4a). We also observed representative Kyoto Encyclopedia of Genes and Genomes (KEGG) pathways that contained metabolic pathways, focal adhesion, MAPK signaling pathway, PPAR signaling pathway, fatty acid metabolism, Calcium signaling pathway, VEGF signaling pathway and other upstream and downstream pathways of HIF signaling (Fig. 3c, Table S4b). In addition, eight DEGs, namely *HSPB1* (heat shock protein family B member 1), *PRRX1* (paired related homeobox 1), *NRXN1* (neurexin 1), *NGFR* (nerve growth factor receptor), *IL8* (interleukin 8), *ACE2* (angiotensin I converting enzyme 2), *LVRN* (laeverin) and *CASQ2* (calsequestrin 2) are mainly associated with angiogenesis, blood circulation, circulatory system development, VEGF signaling pathway, and MAPK signaling pathway. Two DEGs, *TF* (transferrin) and *CACNA1H* (calcium voltage-gated channel subunit alpha1 H), are associated with ion transport. Two DEGs, *SCD* (stearoyl-CoA desaturase) and *NR4A3* (nuclear receptor subfamily 4 group A member 3), are related to fatty acid metabolism, cellular respiration, oxidation–reduction process, and PPAR signaling pathway. Based on the functional annotation, these 10 DEGs may play important roles in adaptation to hypoxic environments in chicken embryos (Table 1).

**Table 1:** Potential key differentially expressed genes (DEGs) and their functions related to hypoxic adaptation in the Tibetan chicken.

	Gene symbol	Log_2_FC of TC/CH in transcriptome	*P *value in transcriptome	FC of TC/CH in proteome	*P *value in proteome	Functional analysis
Angiogenesis, blood circulation	HSPB1	1.35878	3.20E−11	1.337911	0.01458	Blood vessel morphogenesis, vasculature development, angiogenesis, VEGF signaling pathway, MAPK signaling pathway, response to wounding
	PRRX1	2.9814	3.22E−05			Blood vessel morphogenesis, vasculature development, angiogenesis, circulatory system development, embryonic morphogenesis
	NRXN1	1.15815	0.007762			Blood vessel morphogenesis, vasculature development, angiogenesis, circulatory system development
	NGFR	1.0405	0.003605			Blood vessel morphogenesis, vasculature development, angiogenesis, response to oxygen-containing compound, inflammatory response
	ACE2	− 2.81573	0.004862			Regulation of blood circulation, ion transport
	IL8	1.27992	0.000488			Inflammatory response, blood vessel morphogenesis, vasculature development, NOD-like receptor signaling pathway
	LVRN	3.37668	0.004667			Regulation of blood circulation
	CASQ2	2.66033	0.000188			Regulation of blood circulation
	HK2	0.518702	0.013336	1.238302	0.04188	HIF-1 signaling pathway, insulin signaling pathway, ATP binding, reactive oxygen species metabolic process
	PPP1CB	0.009335	0.957726	1.271042	0.01563	Vascular smooth muscle contraction, focal adhesion, insulin signaling pathway, regulation of glucose metabolic process
	Notch2	0.581222	0.00163	1.356528	0.02125	Notch signaling pathway
	NUMB	0.275351	0.128049	1.246141	0.02506	Notch signaling pathway, respiratory system development
	NLN	-0.2943	0.202168	1.359688	0.03799	Renin-angiotensin system, regulation of glucose metabolic process
	AGT	0.760166	0.237922	0.833058	0.00249	Renin-angiotensin system, blood circulation, regulation of blood pressure, vascular smooth muscle contraction
	CTSA	0.174747	0.347873	0.806236	0.00921	Renin-angiotensin system
	EMP2	− 0.31835	0.085554	0.828718	0.01851	Blood vessel development, angiogenesis, blood circulation, regulation of blood pressure
	ALB	− 0.70844	0.181368	0.654163	0.04703	Oxygen binding
	MLST8	0.17267	0.38606	1.402026	0.01272	mTOR signaling pathway, PI3K-Akt signaling pathway
	VAV2	0.005034	0.97868	1.241425	0.01637	Focal adhesion, cAMP signaling pathway
	PPP1R12A	− 0.385	0.077303	0.68122	0.02216	Vascular smooth muscle contraction, Focal adhesion, cAMP signaling pathway
	STK4	0.06014	0.736915	1.289501	0.02507	Angiogenesis, blood vessel development, MAPK signaling pathway, Ras signaling pathway, pathways in cancer
	NCALD	0.12793	0.763732	1.236695	0.01668	Blood circulation, regulation of blood pressure
	PLCB1	− 0.06643	0.793429	0.691824	0.02702	Vascular smooth muscle contraction, calcium signaling pathway, Wnt signaling pathway, pathways in cancer
	CKI	0.010574	0.9535	1.431694	0.04621	Angiogenesis, blood vessel development, carbohydrate metabolic process
	APOH	0.765486	0.313575	0.489209	0.0491	Angiogenesis, blood vessel development
	GNG2	0.167516	0.608336	1.342209	0.00533	Angiogenesis, blood vessel development, PI3K-Akt signaling pathway, Ras signaling pathway, pathways in cancer
Ion transport	TF	1.25436	9.22E−11	1.317705	0.04102	Ion transport, HIF-1 signaling pathway
	CACNA1H	1.18814	0.002184			Ion transport
Energy metabolism	SCD	1.07335	0.003772			Fatty acid metabolism
	NR4A3	1.69574	5.53E−09			Fatty acid oxidation, cellular respiration, embryonic morphogenesis
	FABP3	− 0.12569	0.506738	0.826003	0.01895	PPAR signaling pathway, fatty acid metabolic process
	AKT2	0.071846	0.783787	1.259509	0.02436	ATP binding, regulation of fatty acid oxidation, ion transport, regulation of glucose metabolic process
	IDH3B	0.089861	0.744915	1.242099	0.02527	NAD binding, cellular respiration
	SIRT5	0.087213	0.737814	1.380089	0.00174	NAD binding, reactive oxygen species metabolic process

### Protein identification and quantification

**Figure 4:**
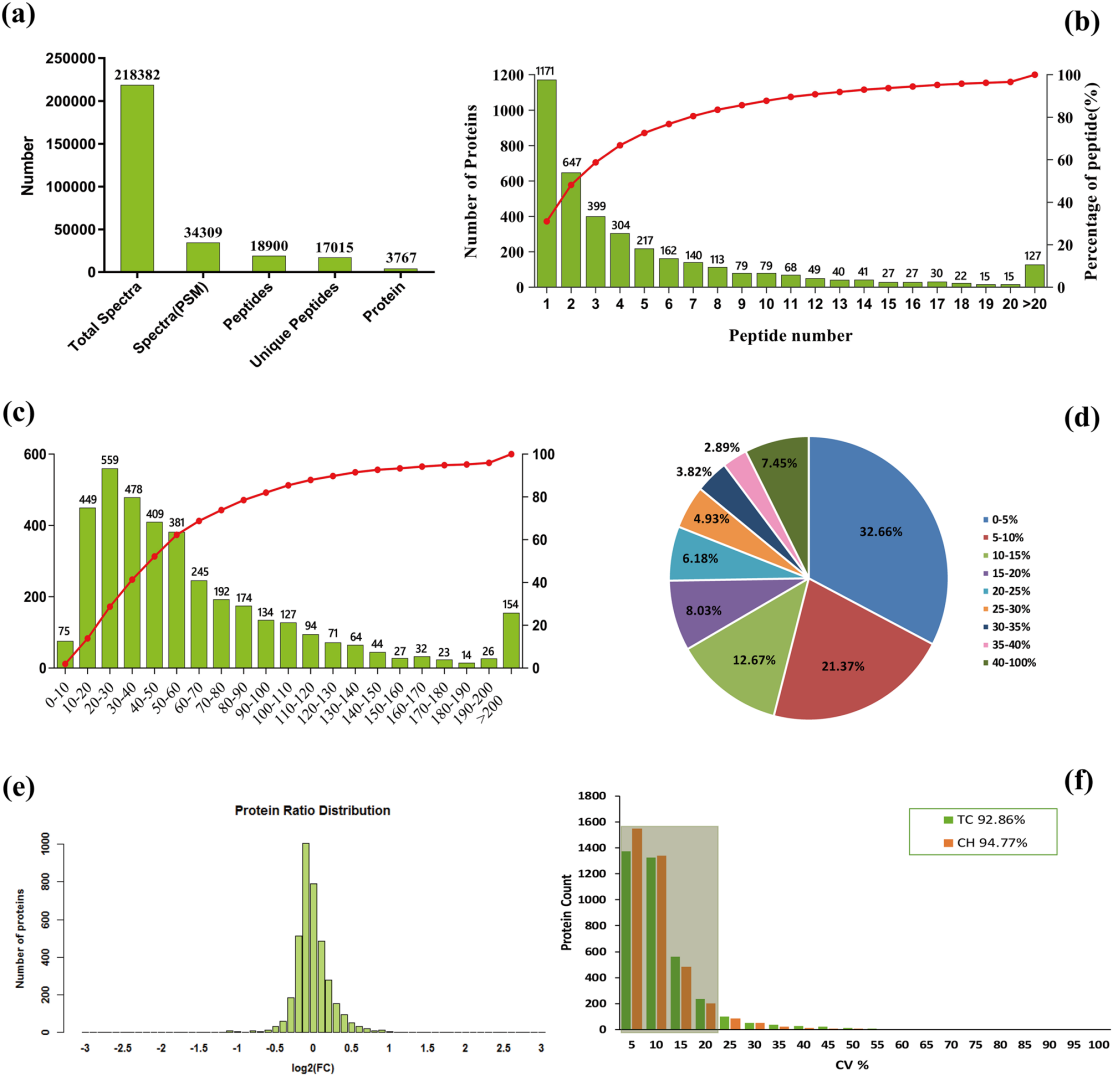
Overview of protein identification information. (**a**) Basic information on proteins identification, (**b**) distribution of proteins containing different numbers of identified peptides, (**c**) distribution of the identified proteins among the different molecular weight classes, (**d**) coverage of proteins by the identified peptides, (**e**) distribution of protein abundance ratio, (**f**) the coefficient of variation (CV) of proteins in the replicates of the two groups.

Using the 6 Plex LC–MS/MS, 218,382 spectra were detected, and 18,900 peptides were obtained (Table S5), in which, 17,015 unique peptides that corresponding to 3,767 proteins (Table S6) were identified (Fig. 4a). On the proteins identified, 72.59% were represented by 1–5 peptides (Fig. 4b), and the protein molecular weight was mainly distributed between 10 and 80 kDa (71.92%) (Fig. 4c). The majority of the proteins were identified with high peptide coverage (Fig. 4d), and the fold changes of the TC/CH were mostly close to 1 (Fig. 4e), indicating that good sequence coverage of the proteins was identified. The proportion of proteins with a variation coefficient less than 20% accounted for over 90%, demonstrating good biological reproducibility within the group (Fig. 4f).

### Identification and functional analysis of differentially expressed protein (DEPs)

**Figure 5:**
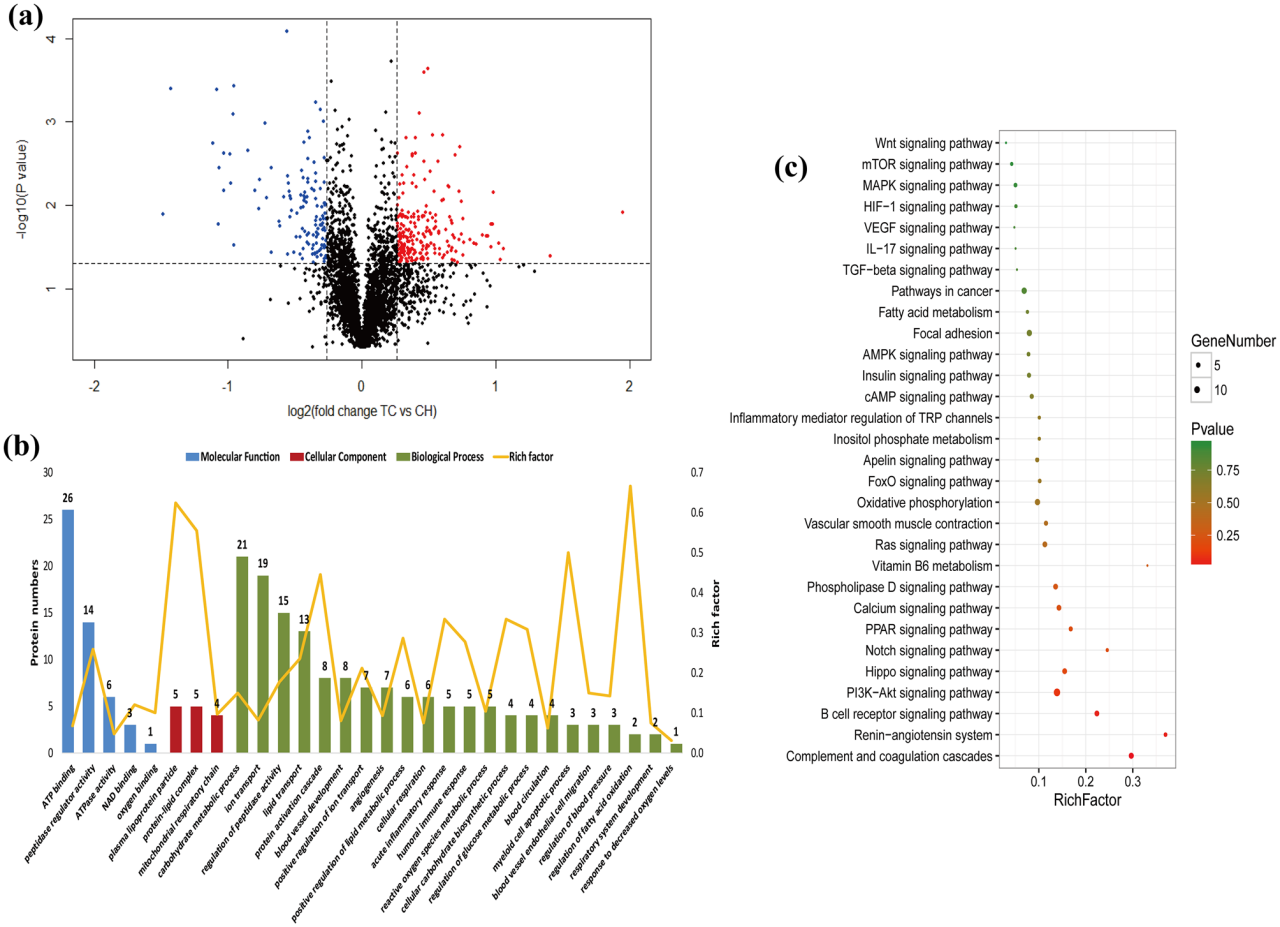
Identification and functional analysis of DEPs. (**a**) Volcano plot displaying DEPs between TC and CH. Genes showing high and low expression in Tibetan chickens are shown in red and blue, respectively. Black dots represent genes with similar expression levels. (**b**) GO enrichment analysis of DEPs between TC and CH. (**c**) KEGG-enriched scatter plot of DEPs between TC and CH^56^. The rich factor is the ratio of DEP numbers annotated in this pathway term to the total gene numbers annotated in this pathway term. The smaller *P* value indicates higher significance. *TC* Tibetan chicken, *CH* Chahua chicken.

Comparing TC and CH, we detected 387 DEPs including 249 up-regulated and 138 down-regulated in the TC (Fig. 5a, Table S7). The DEPs were classified into two categories with good uniformity within groups and distinct diversity between the two groups, indicating that the selected DEPs were relatively accurate (Fig. S5).

Functional annotation of the 387 DEPs revealed GO terms primarily associated with angiogenesis, blood vessel development, blood circulation, oxygen binding, protein activation cascade, carbohydrate metabolic process and other related biological processes (Fig. 5b, Table S8a). The enriched representative KEGG mainly included HIF-1 signaling pathway, VEGF signaling pathway, renin-angiotensin system, Notch signaling pathway, vascular smooth muscle contraction, fatty acid metabolism and other pathways cascaded with HIF signaling (Fig. 5c, Table S8b). Nineteen DEPs (*HSPB1, HK2, PPP1CB, Notch2, NUMB, MLST8, VAV2, ALB, EMP2, AGT, CTSA, NLN, PPP1R12A, STK4, NCALD, PLCB1, CKI, APOH* and *GNG2*) are mainly associated with angiogenesis, blood vessel endothelial cell migration, blood circulation, vascular smooth muscle contraction, VEGF signaling pathway, HIF-1 signaling pathway, renin-angiotensin system and oxygen binding. The gene of *TF* has iron transport function related to increased hemoglobin oxygen carrying capacity and is involved in HIF-1 signaling pathway. Four DEPs (*FABP3, AKT2, IDH3B* and *SIRT5*) are involved in energy metabolism related to cellular respiration and respiratory system development. Based on the functional annotation, 20 DEPs were identified to play potential roles in adaptation to hypoxic environments in Tibetan chicken embryos (Table 1).

### Validation of DEGs and DEPs

Nine DEGs (*CACNA1H, IL8, RRAD, TF, HSPB1, CCR5, CD4, FABP4* and *NOX4*) were selected to validate the expression differences in RNA-Seq using quantitative real-time PCR (qRT-PCR). Specifically, seven genes showed significantly different expression between the TC and CH (Fig. S6a). Further, fold changes of all nine genes in the qRT-PCR and RNA-seq showed the same trends (Fig. S6b). The results indicated that the DEGs identified in RNA-Seq were reliable and efficient.

To further verify the DEPs identified via iTRAQ, 20 proteins were selected to measure expression levels using liquid chromatography parallel reaction monitoring/mass spectrometry analysis (LC-PRM/MS). The results indicated that six proteins were markedly up-regulated and 14 proteins down-regulated in the TC compared with CH, and the expression changes of all 20 proteins were consistent with fold changes in iTRAQ (Fig. S7, Table 2).

**Table 2:** Confirmation of DEPs detected in iTRAQ analysis using LC-PRM/MS analysis.

Accession	Fold change (TC/CH) in iTRAQ	*P* value in iTRAQ	Fold change (TC/CH) in PRM	*P* value in PRM
A0A1D5P4L7	1.32	0.0410	1.29	0.0424
A0A1D5PTS3	1.27	0.0227	1.69	0.0387
B3Y932	1.31	0.0243	2.09	0.0208
E1BZ05	1.49	0.0456	1.57	0.0464
F1NPJ8	1.43	0.0123	1.69	0.0900
R4QXY1	2.65	0.0409	1.94	0.0795
A0A1D5NT90	0.76	0.0297	0.60	0.0416
A0A1D5NW68	0.65	0.0470	0.63	0.0477
A0A1D5PE67	0.79	0.0100	0.65	0.0526
A0A1D5PNU2	0.49	0.0491	0.44	0.0943
E1BSJ2	0.73	0.0392	0.58	0.0499
E1BV96	0.79	0.0014	0.44	0.0411
E1C7A7	0.78	0.0205	0.60	0.0791
F1NII7	0.68	0.0013	0.40	0.0976
F1NWN4	0.71	0.0062	0.37	0.0872
F1NX60	0.79	0.0044	0.52	0.0404
P09244	0.83	0.0182	0.58	0.0323
Q01635	0.82	0.0165	0.67	0.0964
Q90633	0.59	0.0260	0.70	0.0379
Q91025	0.51	0.0112	0.57	0.0803

### Integrated analysis of transcriptome and proteome data

Integrating the 14,869 detected genes by RNA-Seq and 3,767 proteins via iTRAQ, 2,764 genes have expression values in both mRNA and protein levels, and the Pearson correlation coefficient of the fold changes of TC/CH between the mRNA and protein expression levels was 0.12 (Fig. S8a). For the 160 DEGs identified by RNA-Seq and 387 DEPs via iTRAQ, seven genes were found to overlap, of which six genes (*HSPB1*, *TF*, *MYH11*, *BHMT*, *CAP2*, and *DES*) had the same direction of changes in comparing TC/CH, and the Pearson correlation coefficient of the fold change in the seven genes was 0.21 (Fig. S8b, Table S9). Two of these six genes (*HSPB1* and *MYH11*) are related to angiogenesis and vascular smooth muscle contraction; the *TF* gene as transferrin participates in the HIF-1α pathway. The gene of *BHMT* is related to acid metabolic process, *CAP2* and *DES* function in cytoskeleton organization. Furthermore, 19 DEGs identified in RNA-Seq had protein expression values in iTRAQ, and the Pearson correlation coefficient of fold change was 0.21 (Fig. S8c). Additionally, 257 DEPs identified in iTRAQ had mRNA expression levels in RNA-seq, and their Pearson correlation coefficient of fold change was 0.112 (Fig. S8d). RNA-based analyses usually do not fully represent protein dynamics^29^, and the results of our study suggest that substantial post-transcriptional regulations might occur for hypoxic adaptation in embryos of the Tibetan chicken.

## Discussion

Tibetan chickens have inhabited the Qinghai-Tibet Plateau for at least 1,000 years, which is currently known as the most ancient domestic chicken population living at high-altitude areas^1^. Given their long history in a hypoxic environment, evolution and natural selection have allowed Tibetan chickens to develop several heritable physiological characteristics to promote blood oxygen-carrying capacity, which include larger heart and lung organs, higher hemoglobin concentration, and blood-oxygen affinity^1,5^. The chicken CAM, used for gas exchange and nutrient transport during embryonic development is very responsive to hypoxia, and its mass and vascularization increase selectively and even lead to visible curling of vessels under hypoxic incubation in lowland chickens, in which abnormally increased blood vessels result in slow blood flow and embryonic death^28^. In this study, we also observed that the VDI levels of Chahua chicken were higher in hypoxic rather than in normoxic conditions after day 10 of incubation, which corresponded with previous study^28^. However, the VDI of the Tibetan chicken had no significant difference between hypoxic and normoxic incubations. The results suggested that the blunted responses to hypoxic condition on CAM angiogenesis of the Tibetan chicken embryos were one of the characteristics of hypoxic adaptation, which might benefit blood flow and oxygen transportation. This blunted response of angiogenesis in the Tibetan chicken might be similar to the adaptive regulation of hemoglobin and nitric oxide in the Tibetans^30^. To understand the mechanism of the blunted response to hypoxia, genome-wide gene expression analysis could provide insights into functional genes for hypoxic adaptation in Tibetan chicken embryos.

Transcriptome and proteome analyses of special tissues are currently effective methods to identify key genes related to a complex trait genome wide. Due to existing post-transcriptional regulations, transcriptome data are not always consistent with corresponding proteomic data^22,31^, and the abundance of mRNA transcripts does not completely correlate with expression level of certain genes^32^. In this study, the correlation between the fold-change (FC) of mRNA and the FC of protein levels was low (0.12), and only seven genes overlapped between the DEGs in RNA-Seq and the DEPs in iTRAQ. Potential post-transcriptional regulation might play roles in the adaptation to hypoxia in Tibetan chickens. Although RNA-based analyses do often not fully represent protein dynamics^29^, relatively abundant expressed genes can be obtained from RNA-seq comparing with iTRAQ (14,869 vs 3,767). Integration of transcriptomic and proteomic profiles can yield a more comprehensive understanding of functional gene expression under hypoxic adaptation traits in Tibetan chickens.

**Figure 6:**
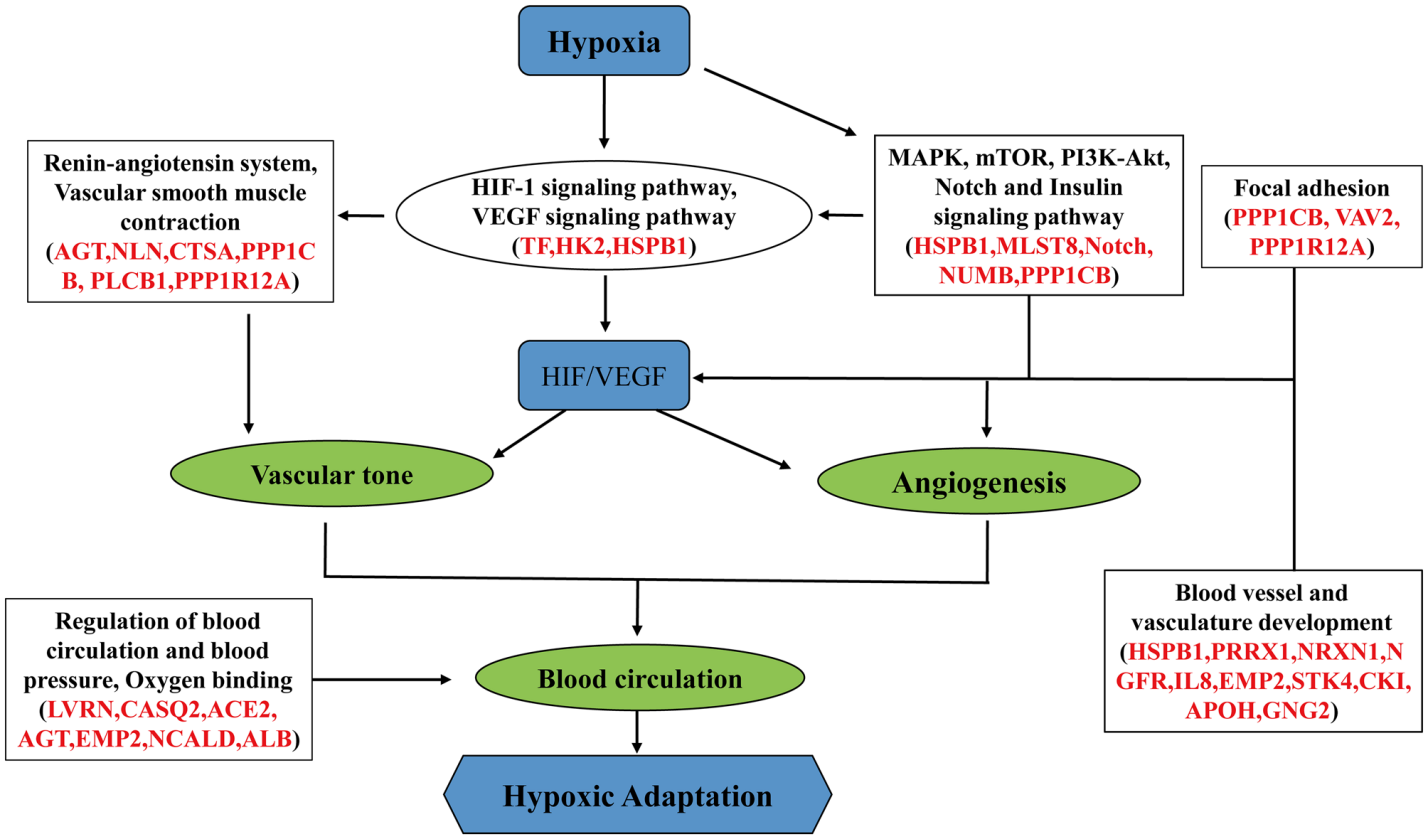
Proposed regulatory mechanisms of blood circulation for hypoxic adaptation in Tibetan chickens. These genes, marked with red color, are involved in HIF-1, VEGF, MAPK, mTOR, PI3K-Akt, Notch, Insulin, focal adhesion signaling pathway, Renin-angiotensin system, vascular smooth muscle contraction and oxygen binding. These pathways and genes indicate Tibetan chickens adapt to hypoxia by regulating angiogenesis and promoting blood circulation (green circles)^56^.

The regulation of angiogenesis is an important component of intravascular homeostasis mechanisms that link vascular oxygen supply to metabolic demand^33^, and are regulated by the VEGF signaling pathway through increased HIF-1 expression^34^. HIF signaling and its surrounding pathways (including VEGF, MAPK, and PI3K-AKT signaling pathways) play key roles in high-altitude adaptation^8,35,36^. Our previous study reported that positively selected genes in highland chicken populations were mainly involved in vasculature development^19^. In the present study, we found HSPB1 (heat shock protein beta-1) and HK2 (hexokinase 2) to be target genes of HIF-1, VEGF and MAPK signaling pathways, and had higher expression in TC than in CH. The blunted response to hypoxia on CAM angiogenesis might be due to upregulation of HSPB1 and HK2 genes in Tibetan chickens. For HIFs and VEGF upstream pathways, MAPK, mTOR, PI3K-Akt, Notch, insulin and focal adhesion signaling pathways and their target genes (*MLST8, Notch, NUMB, PPP1CB, VAV2* and *PPP1R12A*) also contributed to the blunted response to hypoxic conditions on CAM angiogenesis in Tibetan chicken embryos, which could modulate blood vessel development dependent on HIF-1α and VEGF expression^37–40^. Further, we identified a set of associated genes (*PRRX1, NRXN1, NGFR, IL8, EMP2, STK4, CKI, APOH* and *GNG2*) involved in blood vessel and vascular development; coincidentally, a previous study reported the positively selected genes of highland chicken populations mainly enriched in the angiogenesis and blood vessel development functions at the genome level^19^. Our finding, therefore, indicates that Tibetan chickens might modulate angiogenesis by HIF-1 and VEGF signaling pathways and their upstream pathways as well as target gene expression to modify the blunted response to hypoxic condition on the CAM of Tibetan chicken embryos (Fig. 6).

A series of genes, such as *AGT* (angiotensinogen), *CTSA* (cathepsin A), *PLCB1* (phospholipase C beta 1), *PPP1R12A* (protein phosphatase 1 regulatory subunit) and *ALB* (albumin), which are mainly involved in the renin angiotensin system (RAS) pathway, vascular smooth muscle contraction, and oxygen binding, had higher expression in Chahua chickens than in Tibetan chickens under hypoxia. The RAS pathway participates in regulation of vascular tone and has been linked to high-altitude pulmonary edemas^41,42^. It also regulates blood pressure both at the vessel level through vasoconstriction and at the blood level by influencing plasma volume^43^. Tibetan chickens might promote blood circulation by down-regulating these hypoxia-responsive genes. Additionally, three genes (*LVRN*, *CASQ2* and *NCALD*), up-regulated in Tibetan chickens, were related to regulation of blood circulation and blood pressure. Consequently, our findings indicated that Tibetan chickens could improve blood circulation and oxygen bioavailability through the regulation of gene expression involved in angiogenesis and vascular tone in CAM to adapt to hypoxic conditions (Fig. 6).

The CAM also has functions in ion transport that might be involved in hypoxic adaptation, which is responsible for the transport of iron and calcium from the eggshell into the embryo^44^. TF (transferrin), taking part in the HIF-1 signaling pathway, is an iron transport protein that binds to hemoglobin and is involved in the carriage of body oxygen^45^. In addition, Ca^2+^ is necessary for HIF-1 translation, which is considered a key transcriptional regulator of cellular and developmental responses to hypoxia^46,47^. CACNA1H (calcium voltage-gated channel subunit alpha1 H) is a T-type Ca^2+^ channel gene that was previously reported to be associated with the hypoxia response, and its interaction with reactive oxygen species (ROS) plays key roles in hypoxic pulmonary vasoconstriction^48,49^. In our study, the TF and CACNA1H expression levels in Tibetan chickens were higher than in Chahua chickens, suggesting that Tibetan chickens might have adapted to hypoxic conditions through iron and calcium transport involved in up-regulating these genes.

Under hypoxic conditions, oxygen and carbon dioxide metabolism in the CAM mainly depend on mitochondrial respiratory and carbon metabolism and requires adenosine triphosphate (ATP) consumption. Our study also found several functional enrichments of energy metabolism pathways, such as regulation of the glucose metabolic process, carbon metabolism, ATP binding, mitochondrial respiratory chain and fatty acid metabolism. Oxidation of fatty acids yields less ATP per molecule of oxygen consumed than carbohydrate oxidation, suggesting that decreased fatty acid oxidation could also be a favorable adaptation to hypoxia^50^. Therefore, the CAM of Tibetan chickens might have adapted to hypoxic conditions by promoting glucose oxidation and glycolysis and decreasing fatty acid oxidation. We identified several genes (*SCD, NR4A3, FABP3, AKT2, IDH3B* and *SIRT5*) that participate in energy metabolism, thus suggesting Tibetan chickens adapted to hypoxic conditions by enhancing the energy metabolism of these regulated genes.

In summary, we found the VDI in the CAM of the Tibetan chicken was lower than that in the Chahua chicken after 10 days of hypoxic incubation. In CAM tissues, 160 DEGs and 387 DEPs were identified between the Tibetan and Chahua chicken. Combination of transcriptomic and proteomic data revealed several key candidate regulators and pathways that might play high-priority roles in the hypoxic adaptation of Tibetan chickens by regulating angiogenesis and promoting blood circulation, thus explaining the blunted responses to hypoxic conditions on CAM angiogenesis in Tibetan chicken embryos. These results provide new insights into the molecular mechanisms of hypoxic adaptation regulatory networks and the understanding of hypoxic diseases. However, further studies are required to confirm the candidate functional genes in regulating hypoxic adaptation in the future.

## Methods

### Animals and samples preparation

We collected eggs of TC and CH from the Experimental Chicken Farm of China Agricultural University (CAU). The eggs were incubated in a hypoxic incubator (13% ± 0.2% O_2_) and normoxic incubator (21% ± 0.2% O_2_), the temperature and humidity of the incubators were 37.8 °C and 60%, respectively. Eighteen CAM samples of Tibetan (n = 9) and Chahua chickens (n = 9) under hypoxic conditions were collected from embryos at day 11 of incubation, immediately frozen in liquid nitrogen, and stored at − 80 °C for sequencing. The experiments and animal care protocol were approved by the animal welfare committee of the State Key Laboratory for Agro-Biotechnology of the China Agricultural University (approval number, XK257), and all methods were performed in accordance with the relevant guidelines and regulations.

### Vessel density index (VDI)

Tibetan and Chahua chickens incubated in the normoxic and hypoxic conditions. On days 6, 10, 14, and 18 of incubation, eggs were removed from the incubator and opened with a needle at the blunt end where the air sac is located. A circular window of 3 cm diameter was created to allow access to the underlying embryo. The air sac membrane was then carefully removed with fine forceps, ensuring that no vessels were disturbed. CAMs were fixed using a methanol:acetone solution (1:1) for 10 min within the egg. A piece of CAM tissue was removed and spread on a glass slide, and covered with a coverslip. Images of CAMs were captured using a microscope (Leica/Wild M3Z, Wetzlar, Germany). The VDI (intersections/mm) was determined from the number of intersections between all discrete blood vessels and three concentric circles having diameters of 2.05, 2.55, and 3.01 mm (total circumference = 23.9 mm)^28^. A pixel image of the concentric circles was drawn on the monitor screen superimposing the tissue image, and measurements were made in three randomly selected areas of one CAM image using the same set of circles. Eight embryos were measured for CAM images and VDI in each group at each time point.

### RNA extraction and RNA-seq

The CAM tissues of embryos at day 11 of incubation were used in RNA-Seq. Total RNA was extracted using RNA pure Tissue Kit (Tiangen Biochemical Technology Beijing Co., Ltd) according to the corresponding manufacturer’s protocol. Six RNA-Seq libraries were constructed, including three Tibetan chickens (TC1, TC2, TC3) and three Chahua chickens (CH1, CH2, CH3) incubated in hypoxic conditions. Each sample was mixed from three individuals in the same breed. The pooled RNA samples were purified with a RNeasy Micro Kit (Cat. #74004; QIAGEN, Venlo, Netherlands) for cDNA library preparation (approximately 3 μg of total RNA). Poly(A) mRNA isolation, first-and second-strand cDNA synthesis, and fragment, connecting adapter, and cDNA library preparation were performed sequentially with the TruSeq RNA Sample Prep Kit (Cat. #RS-122-2002; Illumina, San Diego, CA, USA) following the manufacturer’s protocol^51^. All libraries were sequenced using the Illumina HiSeq 4,000 platform for paired-end 150-bp sequencing.

### Transcriptome data processing

Quality of the raw reads was assessed using FastQC. All reads for which the quality of more than half of the bases was less than 10, as well as all reads that contained more than two Ns or were smaller than 20 bp in length, were eliminated from subsequent analyses. After removal of the adapters, the remaining clean reads were aligned to the chicken reference genome (Gallus gallus 4.75) (ftp://ftp.ensembl.org/pub/release-75/fasta/gallus_gallus/dna/) using Tophat2 (version 2.0.9) software^52^. The expression levels of the mapped genes were estimated by FPKM. The quantification analyses were conducted using the Cufflink (version 2.1.1) program^53^. Differential expression analysis comparing TC and CH was performed with the Cuffdiff. The P-values were adjusted using the Benjamini–Hochberg method. The FC of TC/CH was calculated with the average expression of each group. DEGs were screened on the criteria of |log_2_FC|≥ 1 and *P* ≤ 0.01. All RNA-Seq data were deposited in the Gene Expression Omnibus under accession number GSE126082, link is (https://www.ncbi.nlm.nih.gov/geo/query/acc.cgi?acc=GSE126082).

### Protein extraction, digestion, and iTRAQ labeling

The pooled samples were used in proteomic analyses were the same as in RNA-Seq. Total proteins were extracted from CAM tissues, and protein concentrations were determined with the BCA protein assay reagent (Beyotime Institute of Biotechnology, Shanghai, China). The proteins were digested to peptides with 2 μg trypsin (Promega) in 40 μL DS buffer overnight at 37 °C and collected as a filtrate. For labeling, the peptides were dried using vacuum-centrifugation, reconstituted in 0.5 M TEAB (Applied Biosystems, Milan, Italy), and processed according to the manufacturer’s protocol for 6-plex iTRAQ Reagent kit^22^. In the labeling reaction, samples were incubated for 2 h at room temperature, pooled, and dried using vacuum-centrifugation^22^.

### LC–MS/MS analysis

Experiments were performed on a Q Exactive mass spectrometer coupled to an Easy nLC (Proxeon Biosystems, now Thermo Fisher Scientific). Briefly, 10 μL of each fraction was injected for chromatography-tandem mass spectrometry (LC–MS/MS) analysis. MS data were acquired using a data-dependent top 10 method dynamically choosing the most abundant precursor ions from the survey scan (300–1,800 m/z) for HCD fragmentation. Determination of the target value was based on predictive Automatic Gain Control (pAGC)^54^. Survey scans were acquired at a resolution of 70,000 at m/z 200, and resolution for HCD spectra was set to 17,500 at m/z 200^54^. Normalized collision energy was 30 eV, and the underfill ratio, which specifies the minimum percentage of the target value likely to be reached at maximum fill time, was defined as 0.1%^54^. The instrument was run with peptide recognition mode enabled. Data are available via ProteomeXchange with identifier accession PXD012607.

### Protein identification and quantification

For peptide identification and quantification, MS/MS data were searched against the “uniprot_Gallusgallus_32644_20170116.fasta” file using the MASCOT engine (Matrix Science, London, UK; version 2.2) embedded into Proteome Discoverer 1.4 (Thermo Electron, San Jose, CA.). Protein quantification was based on the total intensity of the assigned peptides. The six labeled sample mixes were used as a reference. Relative quantification of identified proteins was estimated according to the weighted and normalized ratios of uniquely identified peptides using the median ratio in Mascot^55^. DEPs was determined using Student’s *t* test. FC ≥ 1.2 or FC ≤ 0.83 and *P* value ≤ 0.05 were set as thresholds for identifying DEPs.

### Functional annotation of DEGs and DEPs

Functional annotation of DEGs was performed by GO and KEGG using the ‘KOBAS 3.0’ enrichment analysis tool (https://kobas.cbi.pku.edu.cn/anno_iden.php). Functional analysis of DEPs was conducted using the Blast2GO (Version 3.3.5) program against the GO database (https://geneontology.org/), and the KEGG database (https://geneontology.org/) was used to classify and group the identified proteins.

### Verification of DEGs from RNA-seq

To confirm the DEGs identified in the RNA-Seq, qRT-PCR was carried out on nine DEGs to measure gene expression in embryonic CAM of chickens. The nine individual samples in the RNA-Seq were used in the qRT-PCR. HPRT (hypoxanthine guanine phosphoribosyl transferase) was set as a reference control, and all reactions were run in triplicate. The primer information is listed in Table S10. Fast Quant RT Kit (with gDNase) (Tiangen Biotech Co. Ltd., Beijing, China) was used to synthesize the first-strand cDNA. The qRT-PCR was performed using SuperReal PreMix Plus (SYBR Green) (FP204; Tiangen Biotech Co. Ltd., Beijing, China) on the CFX96 Real-Time System (BIO-RAD, Hercules, CA, USA)^21^. Gene expression levels were calculated using the 2^−△△Ct^ method.

### Verification of DEPs from iTRAQ

To further verify the protein expression levels quantified via iTRAQ analysis, additional quantification through LC-PRM/MS was applied. The proteins (60 μg) from CAM tissues of embryos at day 11 of incubation were prepared, reduced, alkylated, and digested with trypsin following the iTRAQ experimental protocol. The obtained peptide mixtures were introduced into the mass spectrometer via a C18 trap column. The LC-PRM/MS was performed by Q-Exactive HF mass spectrometer (Thermo Fisher Scientific, San Jose, CA) with the same 90 min LC gradient setting as above. Peptide probabilities were calculated by the Percolator algorithm in Proteome Discover, and the false discovery rate was set to 0.01. The analysis of raw data was realized via Skyline (version 3.1.0), and six most intense product ions of the target precursors matching the library were selected as transitions for quantitative data processing and proteomic analysis. The sum peak area of ions was used for quantification. Six biological replicates were included in each breed in the LC-PRM/MS.

### Statistical analysis

Differences in VDI among the groups at each time point were analyzed using an ANOVA test via SPSS 25.0. Differences in gene expressions in the qRT-PCR and the LC-PRM/MS between the two chicken breeds were performed using a *T* test. The results were expressed as mean ± SE, and *P* ≤ 0.05 was considered statistically significant.

## Supplementary information

Supplementary figures

Supplementary table S1

Supplementary table S2

Supplementary table S3

Supplementary table S4

Supplementary table S5

Supplementary table S6

Supplementary table S7

Supplementary table S8

Supplementary table S9

Supplementary table S10
