# Supplementary material for: Comparative transcriptomic and proteomic analyses provide insights into functional genes for hypoxic adaptation in embryos of Tibetan chickens
Source: Sci Rep. 2020 Jul 8;10:11213. doi: 10.1038/s41598-020-68178-w (PMC7343830; doi:10.1038/s41598-020-68178-w)
Supplement: Supplementary file 1 — Supplementary figures [file 41598_2020_68178_MOESM1_ESM.pdf]

Comparative transcriptomic and proteomic analyses provide insights into functional genes for hypoxic adaptation in embryos of Tibetan chickens

Ying Zhang[1], Xiaotong Zheng[1], Yawen Zhang, Hongliang Zhang, Xuyuan Zhang, Hao Zhang*

National Engineering Laboratory for Animal Breeding, Beijing Key Laboratory for Animal Genetic Improvement, College of Animal Science and Technology, China Agricultural University, Beijing, China

*Corresponding author: zhanghao827@163.com

[1] These authors contributed equally to this work.

Figure S1

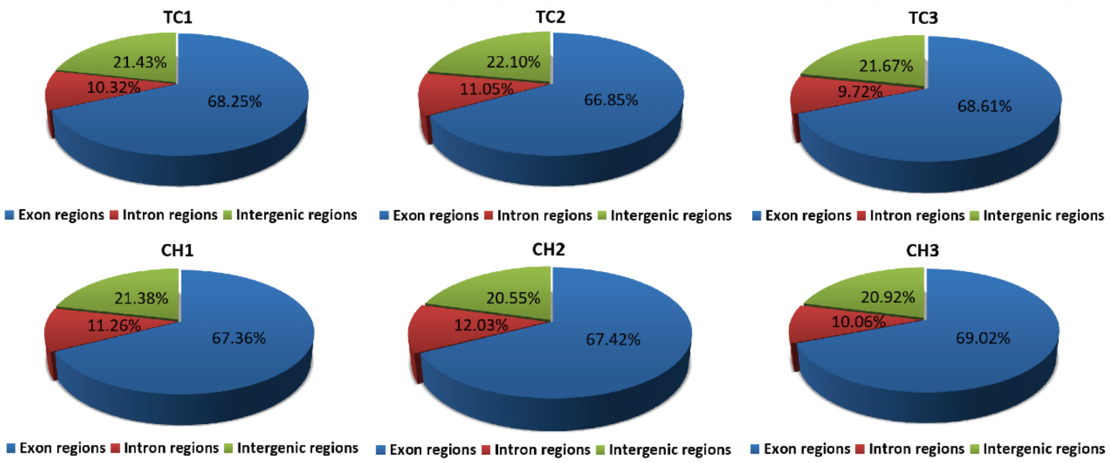

Figure S1. Distribution of clean reads in the chicken genome. Different color represents different distribution region of clean reads. TC1, TC2 and TC3 were Tibetan chicken samples. CH1, CH2 and CH3 were Chahua chicken samples.

Figure S2

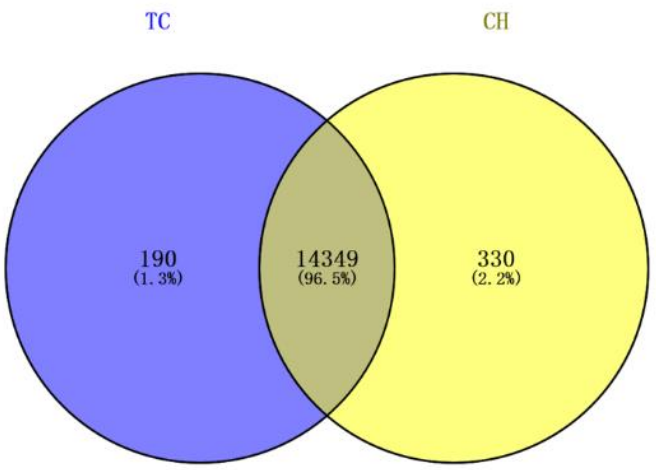

Figure S2. Venn diagrams of the number of genes expressed in two group. TC was Tibetan chicken, CH was Chahua chicken.

Figure S3

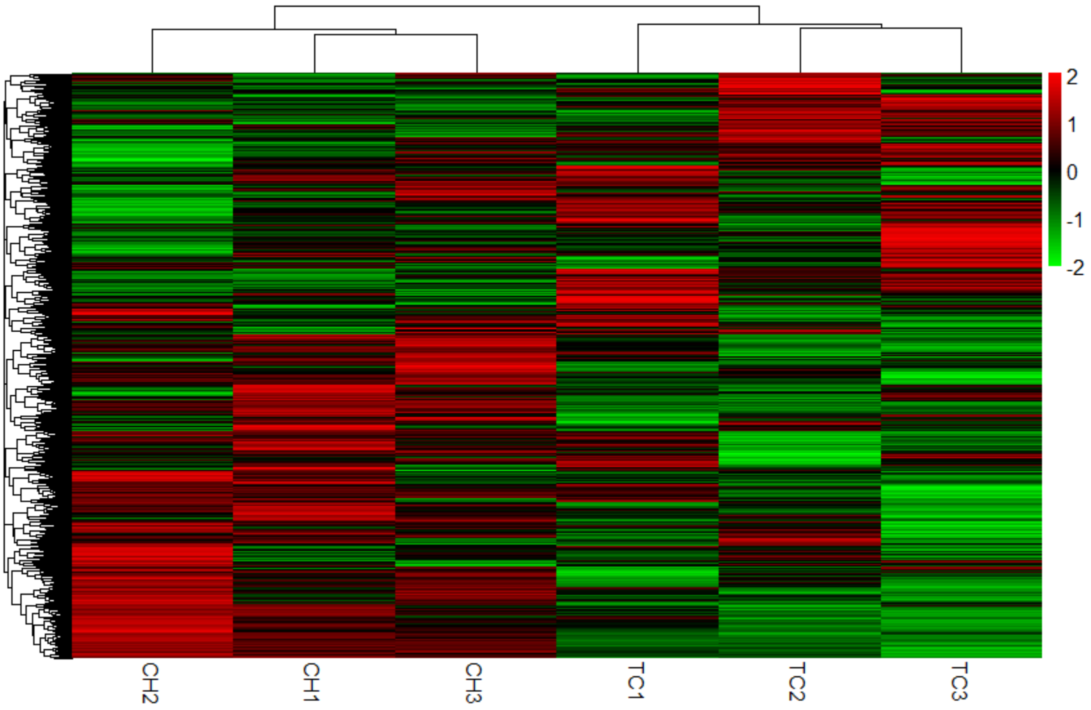

Figure S3. Figure of hierarchical clustering with FPKM of all overlapping genes in Tibetan and Chahua chickens. X and Y coordinates represented samples and genes, respectively. Clustering with log10 (FPKM), red shows high-expressed genes, green shows low expressed genes, color from red to green shows log10(FPKM) from high to low. TC1, TC2 and TC3 were Tibetan chicken samples. CH1, CH2 and CH3 were Chahua chicken samples.

Figure S4

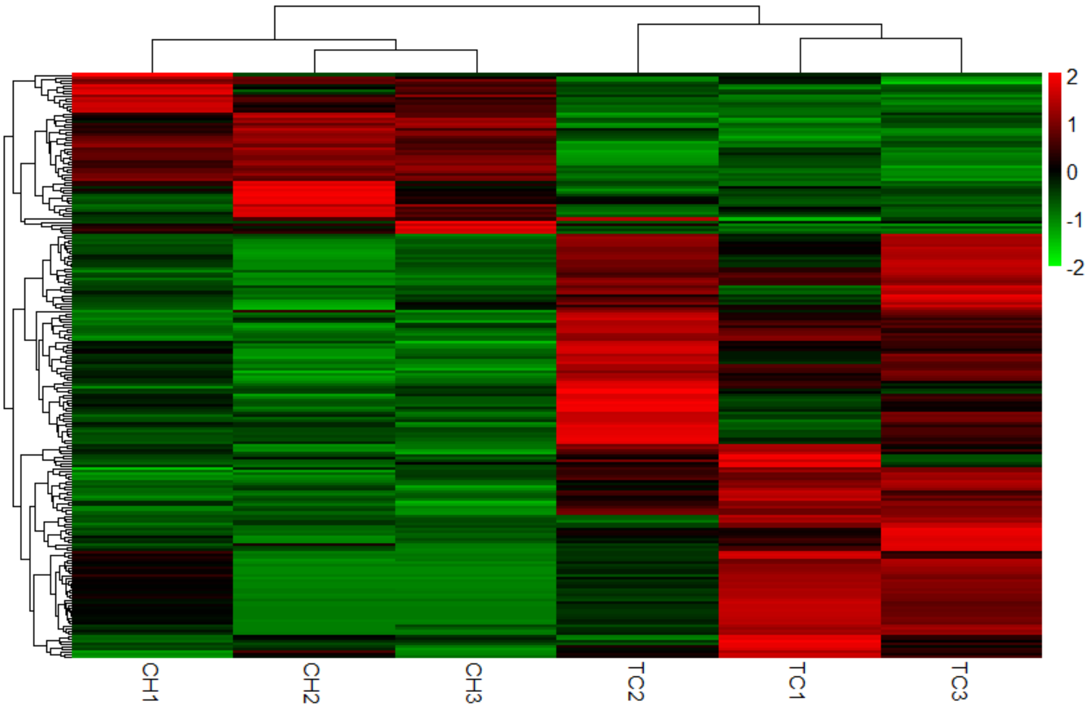

Figure S4. Figure of hierarchical clustering with FPKM of 160 differentially expressed genes in Tibetan and Chahua chickens. X and Y coordinates represented samples and differentially expressed genes, respectively. Clustering with log10 (FPKM), red shows high-expressed genes, green shows low-expressed genes. Vertical comparison indicated that differentially expressed genes could be classified into two categories with opposite directional variation. TC was Tibetan chicken, CH was Chahua chicken.

Figure S5

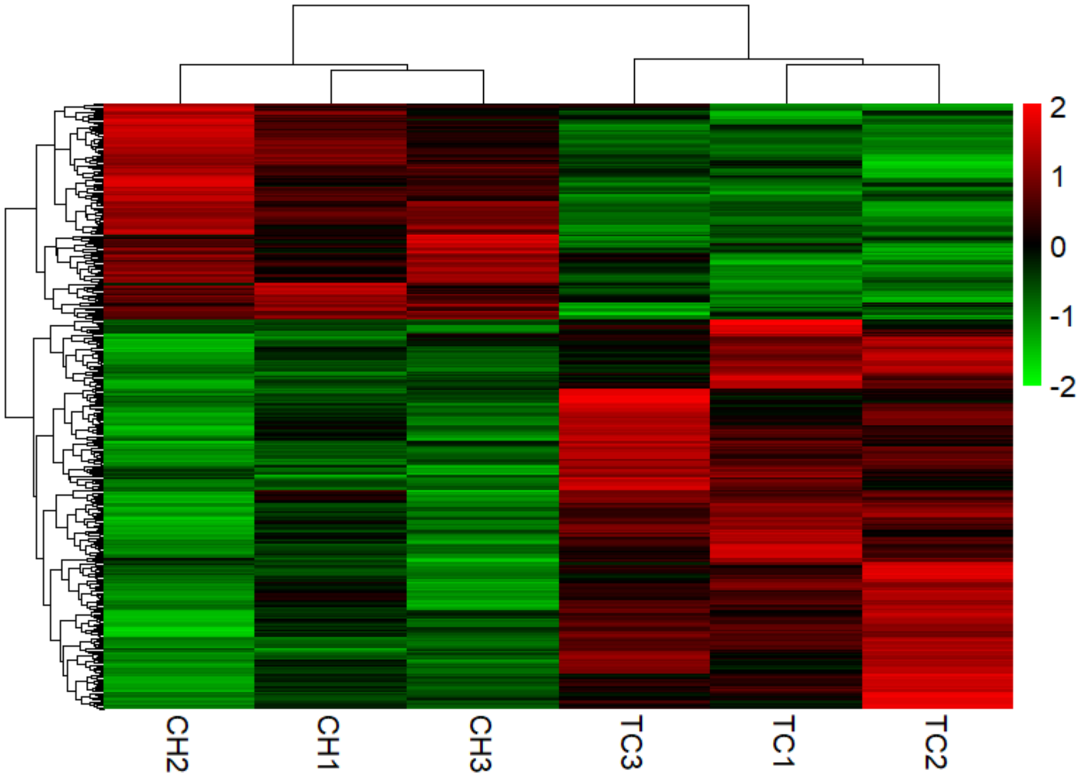

Figure S5. Figure of hierarchical clustering of 387 differentially expressed proteins in Tibetan and Chahua chickens. X and Y coordinates represented samples and differentially expressed proteins, respectively. Red shows high-expressed proteins, green shows low-expressed proteins. Vertical comparison indicated that differentially expressed proteins could be classified into two categories with opposite directional variation. TC was Tibetan chicken, CH was Chahua chicken.

Figure S6

(a)

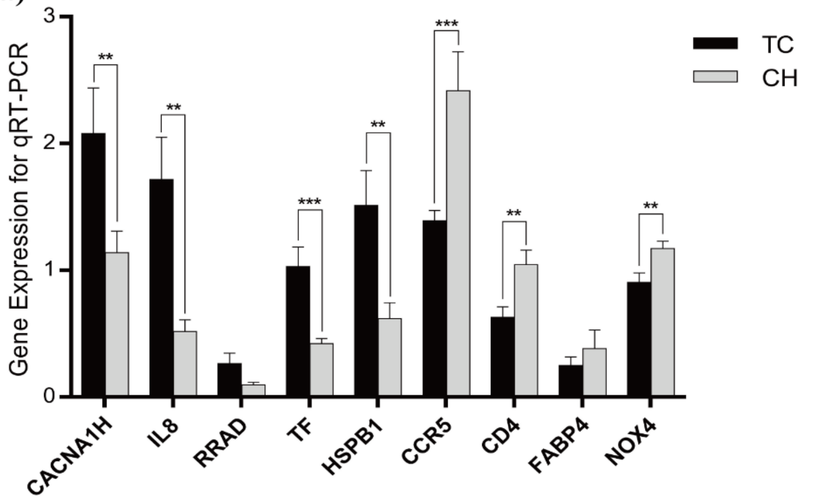

(b)

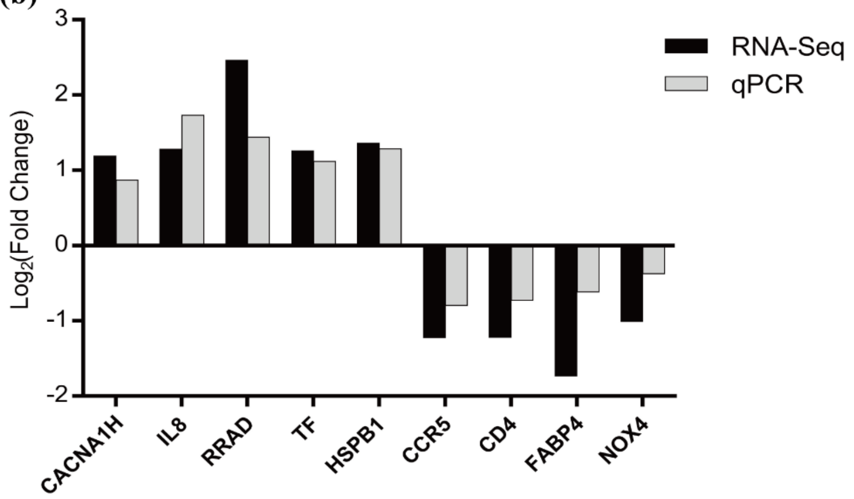

Figure S6. Validation of DEGs by qRT-PCR. (a) The expression of nine genes validated by qRT-PCR in chicken embryo chorioallantoic membrane (CAM) tissue. Note: The vertical axis represents the expression value of mRNA in chicken embryo CAM and the horizontal axis represents names of nine genes. Error bars represent SE of expression. **on the bars indicate significant differences ($P < 0.05$) and *** indicate extremely significant differences ($P < 0.01$) between Tibetan and Chahua chicken breeds. TC = Tibetan chicken (n = 8). CH = Chahua chicken (n = 8). (b) RT-qPCR verification of genes in RNA-seq. The fold changes of the nine genes showed that RT-qPCR results were consistent with the RNA-seq data.

Figure S7

(a)

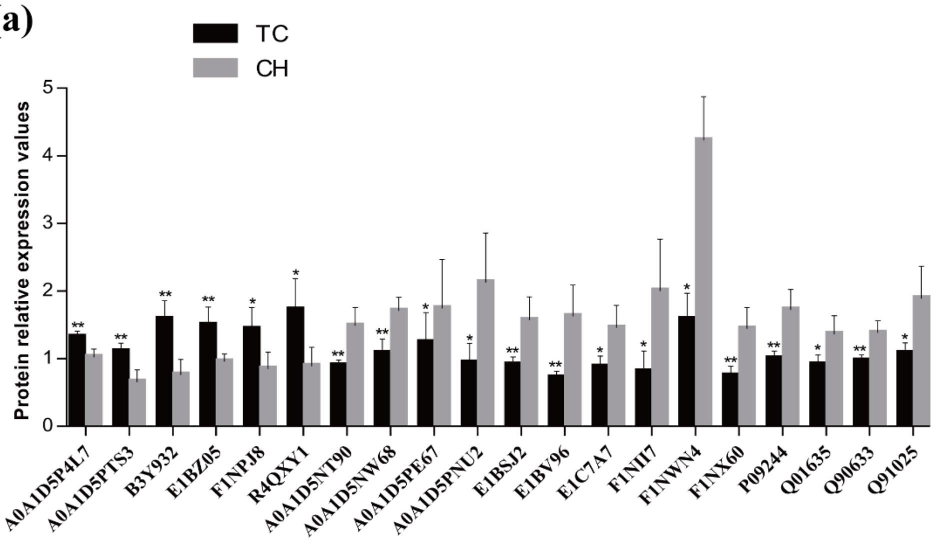

(b)

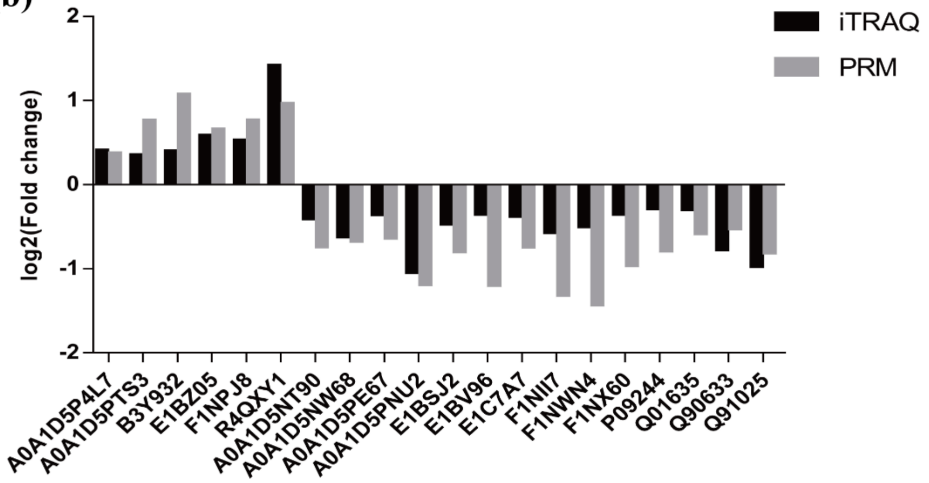

Figure S7. Validation of DEPs by LC-PRM/MS. (a) Protein relative expression values in the PRM experiment. **represents $P < 0.05$, * represents $P < 0.1$ (n = 6 animals of each breed). TC was Tibetan chicken, CH was Chahua chicken. (b) PRM verification of proteins in iTRAQ. The fold change of the 20 proteins showed that PRM results were consistent with the iTRAQ data.

Figure S8

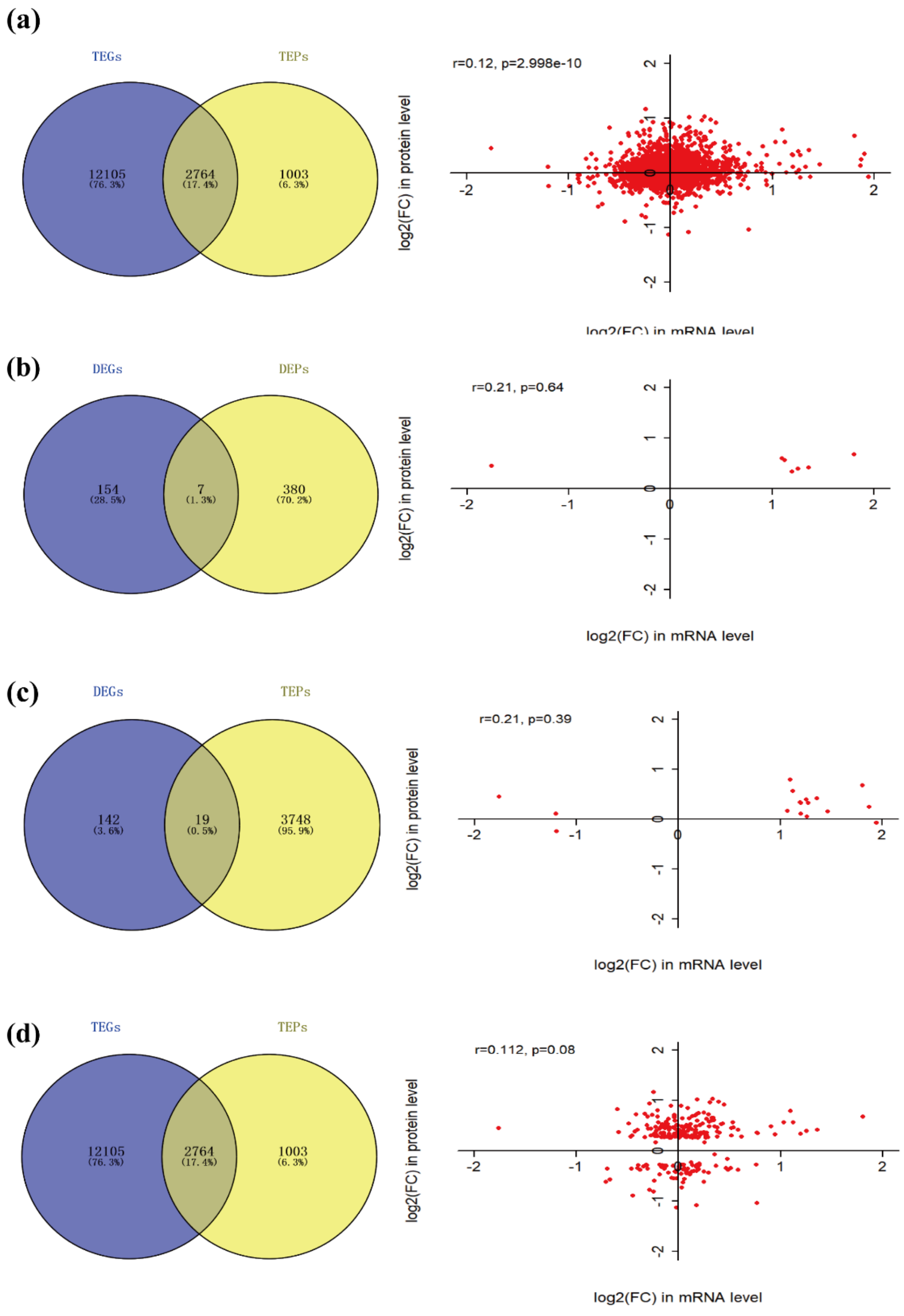

Figure S8. Integrated analysis of Transcriptome and Proteome Data. TEGs: Total expressed genes; TEPs: Total expressed proteins; DEGs: Differentially expressed genes; DEPs: Differentially expressed proteins.
